# Alterations of local functional connectivity in lifespan: A resting‐state fMRI study

**DOI:** 10.1002/brb3.1652

**Published:** 2020-05-27

**Authors:** Xin Wen, Hui He, Li Dong, Junjie Chen, Jie Yang, Hao Guo, Cheng Luo, Dezhong Yao

**Affiliations:** ^1^ College of Information and Computer Taiyuan University of Technology Taiyuan China; ^2^ The Clinical Hospital of Chengdu Brain Science Institute MOE Key Lab for Neuroinformation University of Electronic Science and Technology of China Chengdu China; ^3^ School of Life Science and Technology University of Electronic Science and Technology of China Chengdu China

**Keywords:** four‐dimensional spatial‐temporal consistency of local neural activity, lifespan, local functional connectivity, local functional connectivity density, resting‐state fMRI

## Abstract

**Introduction:**

As aging attracted attention globally, revealing changes in brain function across the lifespan was largely concerned. In this study, we aimed to reveal the changes of functional networks of the brain (via local functional connectivity, local FC) in lifespan and explore the mechanism underlying them.

**Materials and Methods:**

A total of 523 healthy participants (258 males and 265 females) aged 18–88 years from part of the Cambridge Center for Ageing and Neuroscience (CamCAN) were involved in this study. Next, two data‐driven measures of local FC, local functional connectivity density (lFCD) and four‐dimensional spatial‐temporal consistency of local neural activity (FOCA), were calculated, and then, general linear models were used to assess the changes of them in lifespan.

**Results:**

Local functional connectivity (lFCD and FOCA) within visual networks (VN), sensorimotor network (SMN), and default mode network (DMN) decreased across the lifespan, while within basal ganglia network (BGN), local connectivity was increased across the lifespan. And, the fluid intelligence decreased within BGN while increased within VN, SMN, and DMN.

**Conclusion:**

These results might suggest that the decline of executive control and intrinsic cognitive ability in the aging population was related to the decline of functional connectivity in VN, SMN, and DMN. Meanwhile, BGN might play a regulatory role in the aging process to compensate for the dysfunction of other functional systems. Our findings may provide important neuroimaging evidence for exploring the brain functional mechanism in lifespan.

## Graphical abstract

We used local functional connectivity (FCD and FOCA) to explore changes across the lifespan and behavior scores (fluid intelligence and response time in response time tasks). Our findings may provide important neuroimaging evidence for exploring the brain functional connectivity mechanism across the lifespan.

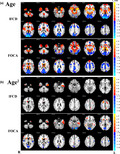

## 1 INTRODUCTION

With the intensification of global aging, lifespan research has become a hot spot (Bookheimer et al., 2019; Taylor et al., 2017; Wei, Zhuang, et al., 2018). Recently, the development and application of functional networks via functional connectivity (FC) have provided new perspectives and discoveries for changes in brain function across the lifespan (Han et al., 2018; Nazeri et al., 2015; Vij, Nomi, Dajani, & Uddin, 2018). In previous behavioral studies (Grady, 2012; Mansson et al., 2015; Smith et al., 2015), it was suggested that with the development of the human brain (young‐mature‐aging), some cognitions and behaviors including motor ability, vision, and auditory would exhibit an inverted U‐shaped curve. Such a phenomenon could be explained that cognition and behavior showed a trend of increasing first and then decreasing with age (Damoiseaux, 2017; Wing et al., 2018). Using functional magnetic resonance imaging (fMRI), several brain function researches indicated that the elderly have lower functional connectivity within the default mode network (DMN), dorsal attention network (DAN), sensorimotor network (SMN), visual (VN) and fronto‐parietal networks (FPN; Betzel et al., 2014; Cassady et al., 2019; Ferreira & Busatto, 2013; Grady, Sarraf, Saverino, & Campbell, 2016; Spreng, Stevens, Viviano, & Schacter, 2016), as well as higher functional connectivity within the basal ganglia network (BGN; Allen, 2011). Meanwhile, structural MRI‐related studies have shown that in addition to temporal lobe‐related networks, older people have lower gray matter volumes in the DMN, DAN, and auditory networks (Damoiseaux, 2017; Liu, 2017). In addition, several previous lifespan and aging studies showed that older people had higher functional connectivity between networks (such as DMN, FPN, DAN, and cingular‐opercular network [CON]; Damoiseaux, 2017; Grady et al., 2016; Wang, Su, Shen, & Hu, 2012; Xia et al., 2019). In general, as the aging process deepens, the functional connectivity within network decreased and the functional connections between networks increased (Ferreira & Busatto, 2013; Vij et al., 2018). Reduced functional connectivity may be due to reduced cognitive and other functions, and the increase in functional connectivity between networks may be due to the compensatory mechanism of the functional network (Damoiseaux, 2017; Ferreira & Busatto, 2013; Naik, Banerjee, Bapi, Deco, & Roy, 2017; Vij et al., 2018). Furthermore, BGN is associated with a variety of functions, including motor, cognitive, motivational, and emotional processes (Doya, 2000), and has overlapping several functions with other networks, especially with SMN which could be expected that BGN may compensate to the dysfunction of SMN (Figley, 2017; Yanagisawa, 2018). Meanwhile, Regners et al. also found that increased effective connectivity from dDMN to BGN in long‐term abstinence may be a compensatory mechanism related to behavioral monitoring (Castellazzi, 2014; Regner et al., 2016). However, the physiological mechanisms of functional network changes in lifespan are still unclear, especially whether and how the potential compensation mechanism is in lifespan.

In recent years, resting‐state fMRI has been used as a powerful tool for exploring spontaneous brain activity and changes in brain function (Biswal, Zerrin Yetkin, Haughton, & Hyde, 1995; De Luca, Beckmann, De Stefano, Matthews, & Smith, 2006). Unlike traditional task fMRI focusing on a single functional system at a time, resting‐state fMRI can provide important spontaneous activity information of functional connectivity for the interpretation of lifespan functional network changes (Betzel et al., 2014; Li et al., 2019; Tomasi & Volkow, 2012; Yang, 2016). Considering that the local functional homogeneity of spontaneous activity is neurobiologically relevant due to possible anatomical, developmental, and neurocognitive factors (Jiang et al., 2015; Wei, Chang, et al., 2018), there are two popular resting‐state measures of local functional connectivity (local FC). One is the local functional connectivity density (lFCD), which reflects the local functional hubs of the brain in the time domain ( Tomasi & Volkow, 2010), and another is the four‐dimensional spatial‐temporal consistency of local neural activity (FOCA), which reflects the functional state of the brain locally in the time–frequency domain (Azeez & Biswal, 2017; Dong et al., 2015). These two local FC measures may comprehensively assess the local functional activity, more comprehensively, from its specific perspective. Therefore, with the advantages of satisfactory reproducibility and reliability, both data‐driven lFCD and FOCA measures of local FC may be suitable for revealing changes in brain function and its mechanism across the lifespan.

This study was the first to explore the alterations of functional networks in lifespan and the possible underlying mechanism through local FCs of lFCD and FOCA, to our knowledge. In this study, we hypothesized that the compensation across the lifespan may exist in a wide range of high‐level (e.g., DMN) and primary networks (e.g., VN and SMN) via local functional connectivity, and BGN may play important role in the compensatory mechanism. Using resting‐state fMRI, lFCD and FOCA were calculated on healthy participants aged 18–88 years. Then, a general linear model (GLM) was used to assess the relationships between local functional indicators and age/age^2^, while controlling the nuisance items of the gender, head motion, and intracranial volume. In addition, relationships between functional measures and behavior scores were also investigated using GLM, while adding the nuisance items as covariates. We predicted that it may provide important neuroimaging evidence for exploring the brain mechanism in lifespan.

## 2 MATERIALS AND METHODS

### 2.1 Participants

In this study, 523 healthy participants (258 males and 265 females) aged 18–88 years from part of the Cambridge Center for Ageing and Neuroscience (CamCAN, http://www.cam‐can.org/; Taylor et al., 2017) were involved. Among all the participants, the proportion of male and female participants remained equally, and there were about 100 participants per 10‐year‐old. Participants performed cognitive tasks outside the MRI scanner. The tests used in this study were the Cattell Culture Fair Test (complete nonverbal puzzles involving series completion, classification, matrices, and conditions), to assess fluid intelligence (Horn & Cattell, 1966), and the speed choice reaction time (RT) task (participants were required to respond as quickly as possible to 1 of 4 possible cued fingers using a 4‐button response box, total trail was 67), to assess speed of processing. For the RT tasks, the mean (M‐RT) and variability (*SD* of RT values, *SD*‐RT) were computed from individual trials. Moreover, due to the lack of records and data quality control, some participants were excluded in this study (see Table 1 and Table S2 for details). All procedures followed the Helsinki Declaration and have been approved by the Cambridgeshire 2 Research Ethics Committee, a local ethics committee in the UK.

**TABLE 1:** Information of participants

	Number	Male/female	Range (years/values)	Mean	*SD*
Age	523	258/265	18–88	51.28	17.60
Cattell	509	254/255	12–44	32.82	6.37
RT_M	476	236/240	0.35–1.11	0.57	0.13
RT_SD	476	236/240	0.04–0.39	0.12	0.06

### 2.2 fMRI data acquisition and preprocessing

Imaging data were collected by a 3T MRI scanner (Siemens TIM Trio). During the scanning period, all participants were required to lie still and keep their eyes closed. The T1‐weighted anatomical images were gathered using an MPRAGE sequence with the following parameters: TR/TE = 2,250 ms/2.99 ms; flip angle = 9°; FOV = 256×240 × 192 mm^3^; voxel size = 1 × 1 × 1 mm^3^. For resting‐state fMRI measurements, 261 volumes of echo‐planar imaging (EPI) sequences were acquired with the following parameters: sequential descending order; slice thickness 3.7 mm with a slice gap of 20% for whole‐brain coverage; TR/TE = 1,970 ms/30 ms; flip angle = 78°; FOV = 192×192 mm^2^; voxel size = 3×3 × 4.44 mm^3^; number of slices = 32; duration time = 520 s.

The resting‐state fMRI data were preprocessed using SPM12 as implemented in the Neuroscience Information Toolbox (NIT, version 1.3, http://www.neuro.uestc.edu.cn/NIT.html; Dong, 2018). The first 5 volumes were deleted from the resting‐state fMRI data of each subject for excluding T1 saturation effects. Then, the images were preprocessed including realignment, slice time correction, spatial normalization using T1‐weighted MRI data (3 × 3 × 3 mm^3^). The head motion of each participant was calculated using the mean framewise displacement (mean FD; Power, Barnes, Snyder, Schlaggar, & Petersen, 2012). Participants whose FD was two or more *SD* above the group mean FD were excluded from further analysis.

### 2.3 lFCD and FOCA

In this work, lFCD and FOCA measures were calculated to assess the local brain activity in resting state using NIT software (http://www.neuro.uestc.edu.cn/NIT.html, version 1.3; Dong, 2018). The local functional connectivity density (lFCD) reflects the distribution of functional hubs of the human brain from the perspective of time consistency in the local region (Tomasi & Volkow, 2010). For a voxel *v* in the brain, the lFCD value of voxel *v* was the number of voxels in the contiguous functional connectivity cluster in a local region (Tomasi & Volkow, 2010). The Pearson correlation was used to assess the strength of the functional connectivity between voxel *v* and its closest neighbors of voxels functionally connected, and a given time courses threshold of 0.6 and the MRI signal‐to‐noise ratio threshold of 50 were used to ensure significant functional connectivity (Tomasi & Volkow, 2010). A voxel was added to the list of voxels connected with voxel *v* only if it was linked to voxel *v* by a continuous path of connected voxels and correlation was larger than the given threshold and this computation repeated for the next voxel in the list until no new neighbors can be added to the list. This calculation was performed for all voxels, and the individual map was obtained by dividing by the mean value of the map. Before calculating lFCD maps, normalized functional images were passband‐filtered (0.01–0.08 Hz) and nuisance signals, including 12 head motion parameters (6 parameters of translation and rotation and their derivative), linear trend, global mean, individual mean white matter, and cerebrospinal fluid signals, were removed from the unsmoothed fMRI data.

On the other hand, the four‐dimensional spatial‐temporal consistency of local neural activity (FOCA) reflects the functional state of the brain locally consist in time–frequency domain (the consistency from both temporal homogeneity of local adjacent voxels using temporal correlation and regional stability of brain activity states between neighboring time points; Azeez & Biswal, 2017; Dong et al., 2015). For each voxel, FOCA value was calculated as mean temporal and spatial correlation of a local region from normalized functional images. After calculating FOCA of all voxels across the brain, the FOCA value was divided by the mean value of the whole brain. Brain regions with high FOCA values are considered to have higher consistency of local spontaneous activity. Before calculating FOCA maps, nuisance signals, including 12 head motion parameters, linear trend, individual mean white matter, and cerebrospinal fluid signals, were removed from the unsmoothed fMRI data. The detailed information of FOCA can be seen in Dong et al. (2015). Finally, the lFCD image and FOCA image were smoothed with an 8 mm FWHM Gaussian kernel.

### 2.4 Statistical analysis

To exhibit the relationship between local functional connectivity and age, we used GLM to fit local measures (lFCD and FOCA maps) over age/age^2^ while adding the gender (*X*_sex_), head motion (mean framewise displacement, *X*_mFD_), and intracranial volume (*X*_volume_) as covariates (Equation 1). Additionally, GLM was also conducted to fit linear local measures over behavior scores (fluid intelligence and speed choice reaction time) adding the same covariates (*X*_behavior_ in Equation 2). Because the changes of mean FCs across lifespan were mainly linear and quadratic (the inverted U shape; Grady, 2012) and FC changes within some functional networks (VN, CEN) have linear decreases across lifespan while those within DMN have quadratic (the inverted U shape) decreases, and SMN, SAN shown both linear and quadratic decreases of FCs (Vij et al., 2018; Wang et al., 2012). It is reasonable to apply a GLM to reveal the age effect on local FCs. Furthermore, the age and behavioral score were strongly correlated (*r* ^2^ = .43, *p* = 3.6 × 10^–63^, see Figure S1). Because adding the behavioral score in the GLM in Equation 1 would lead to a collinearity problem, according to the previous studies (Kievit, 2014; Onoda, Ishihara, & Yamaguchi, 2012), two GLMs were applied to these two measures, respectively.

(1) $$\text{localFC}=\beta_{0}+\beta_{1}\times \text{age}+\beta_{2}\times {\text{age}}^{2}+\beta_{3}\times X_{\text{sex}}+\beta_{4}\times X_{\text{mFD}}+\beta_{5}\times X_{\text{volume}}$$

(2) $$\text{localFC}=\beta_{0}^{\prime }+\beta_{1}^{\prime }\times X_{\text{behavior}}+\beta_{2}^{\prime }\times X_{\text{sex}}+\beta_{3}^{\prime }\times X_{\text{mFD}}+\beta_{4}^{\prime }\times X_{\text{volume}}$$

For each local functional measure (lFCD and FOCA maps), the T value of coefficients of age and age^2^ in Equation 1 (*β*_1_ and *β*_2_, respectively) was used to measure the impact of age (linear and quadratic) on local functional measures. Meanwhile, the T value of coefficients of behavior score (Cattell score, M_RT, and SD_RT) in Equation 2 ( $\beta_{1}^{\prime }$ ) was used to measure the significance of the linear relationship between them and local functional measures. All the significance level was set to *p* < .05, false discovery rate (FDR)‐corrected.

## 3 RESULTS

### 3.1 Changes of lFCD and FOCA

**FIGURE 1:**
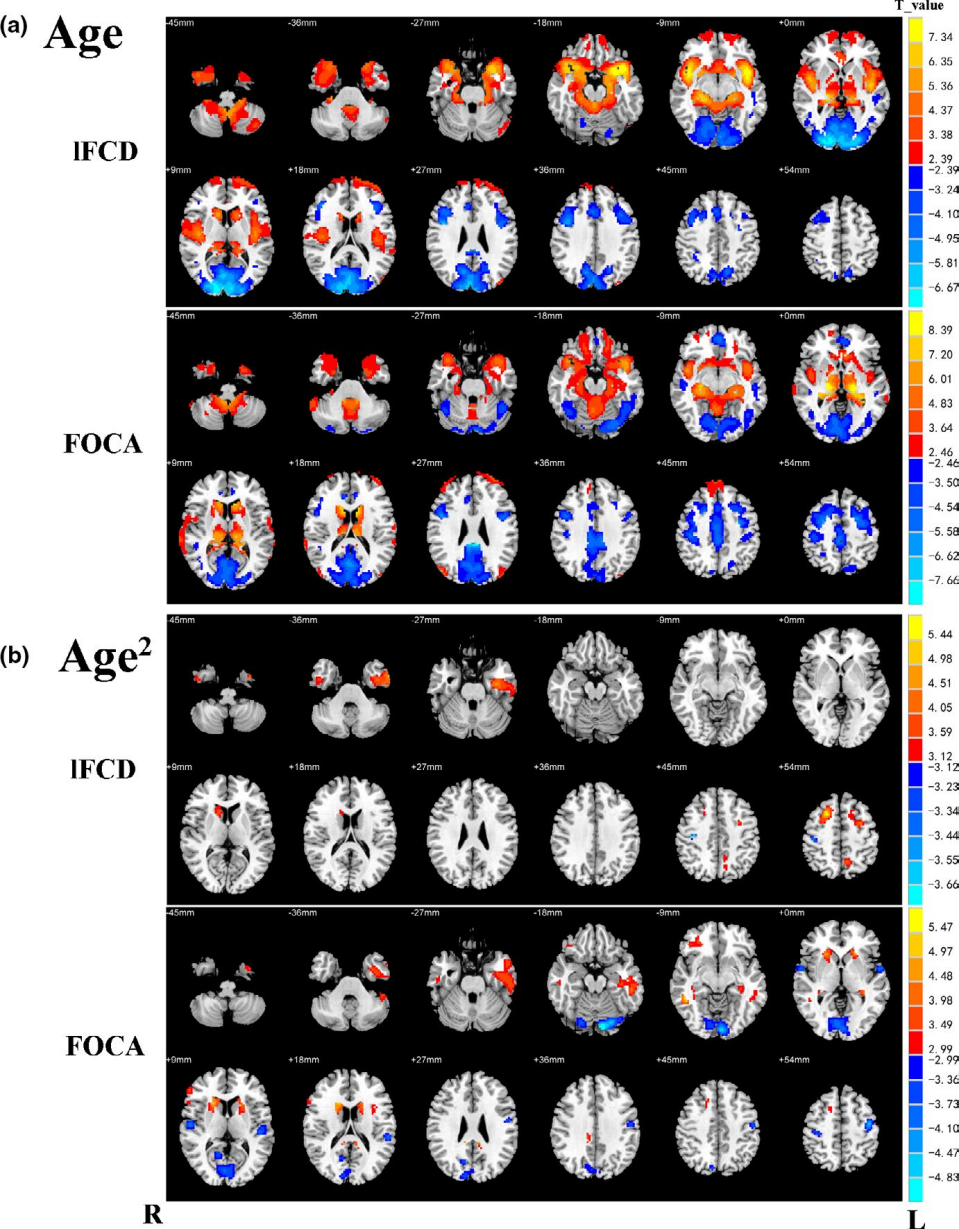
Relationships between local functional connectivity (FOCA and lFCD) and age/age^2^. (a) Impact of age on local FC and (b) impact of age^2^ on local FC (cluster size > 30, *p* < .05, false discovery rate (FDR) correction). Red areas indicate that local FC increased significantly as age increased, while blue areas indicate the opposite. R stands for the right hemisphere of the brain, and L stands for the left hemisphere of the brain

First, we analyzed the linear correlation between local functionality (lFCD and FOCA) and age. During the resting state, the lFCD values in the olfactory cortex, the superior temporal gyrus, right insula, hippocampus, right amygdala, cerebellum inferior, and right caudate nucleus were positively correlated with age, while areas that negatively correlated with lFCD involved occipital gyrus, calcarine, left cuneus, left lingual gyrus, inferior frontal gyrus, left precentral gyrus, and left medial superior frontal gyrus. At the same time, the FOCA values of thalamus, caudate nucleus, hippocampus, superior temporal gyrus, left middle frontal gyrus, and left medial superior frontal gyrus were positively correlated with age; the areas that negatively correlated with FOCA involved right calcarine, right paracentral lobule, right lingual gyrus, cerebellum superior, cerebellum superior, right medial and lateral cingulate gyrus, postcentral gyrus, and left superior orbital frontal gyrus (Figure 1a, detailed information located in Table 2). Meanwhile, results of lFCD with time course thresholding of 0.5 and 0.7 were similar to the above findings and are shown in Figures S2 and S3; and there also were wide changes of degree of global networks at the voxel level in the brain across lifespan (Figure S4).

**TABLE 2:** Detailed information on each brain region for the relationship between local FC and age

Index	Region	MNI coordinates			Peak *t*_value	Cluster size
		*X*	*Y*	*Z*		
*Age*						
lFCD	Olfactory_L	−27	12	−18	8.34	9,204
	Temporal_Pole_Sup_L	−45	9	−12	8.17	
	Temporal_Sup_R	42	6	−12	8.09	
	Insula_R	27	18	−18	7.26	
	Hippocampus_R	24	−30	−3	7.15	
	Amygdala_R	21	3	−12	6.92	
	Temporal_Sup_L	−45	−9	0	6.83	
	Olfactory_R	18	6	−15	6.63	
	Hippocampus_L	−24	−24	−6	6.57	
	Cerebelum_9_L	−9	−45	−42	6.57	
	Cerebelum_9_R	3	−48	−42	6.23	
	Caudate_R	9	18	12	6.17	
	Occipital_Mid_R	15	−93	6	−7.52	4,248
	Calcarine_L	−21	−96	3	−7.13	
	Occipital_Mid_L	−27	−93	0	−6.95	
	Cuneus_R	6	−90	27	−6.48	
	Calcarine_R	3	−75	12	−6.27	
	Lingual_L	−9	−72	−3	−5.57	
	frontal_Inf_Tri_R	39	21	27	−4.92	736
	Precentral_L	−48	6	30	−4.56	434
	Frontal_Sup_Medial_L	0	18	42	−4.62	208
FOCA	Thalamus_L	−18	−24	0	9.32	6,903
	Thalamus_R	21	−21	0	9.57	
	Caudate_L	−9	15	15	8.8	
	Hippocampus_R	24	−30	−3	8.38	
	Caudate_R	9	15	15	8.34	
	Hippocampus_L	−33	−36	0	7.56	
	Temporal_Pole_Sup_L	−45	12	−16	7.23	
	Frontal_Mid_L	−30	60	30	4.36	139
	Frontal_Sup_Medial_L	0	57	45	4	172
	Calcarine_R	3	−66	21	−5.94	7,491
	Paracentral_Lobule_R	3	−33	51	−5.72	
	Lingual_R	9	−75	−3	−5.49	
	Cerebelum_6_L	−27	−78	−18	−5.35	
	Cerebelum_Crus1_L	−45	−54	−24	−5.35	
	Cingulum_Mid_R	3	−15	45	−5.08	
	Postcentral_R	42	−27	48	−4.68	
	Postcentral_L	−45	−27	45	−3.54	902
	Frontal_Mid_Orb_L	0	48	−12	−5.65	258
	Cerebelum_Crus1_R	42	−60	−24	−4.98	231

In addition, a nonlinear correlation between local functionality (lFCD and FOCA) and age was analyzed. The lFCD values in the right dorsolateral superior frontal gyrus, left hippocampus, left precentral gyrus, left precuneus, right inferior temporal gyrus, and right caudate nucleus were positively correlated with age, while area that negatively correlated with lFCD involved right postcentral gyrus. At the same time, the FOCA values of the inferior temporal gyrus, caudate nucleus, and right inferior frontal orbital gyrus were positively correlated with age; the areas that negatively correlated with FOCA involved cerebellum superior, right calcarine, precentral gyrus, superior temporal gyrus, left postcentral gyrus, and transverse temporal gyrus (Figure 1b, detailed information located in Table 3). In addition, gender and age together have few impacts on local FCs in our study (Figure S5).

**TABLE 3:** Detailed information on each brain region for the relationship between local FC and age^2^

Index	Region	MNI coordinates			Peak *t*_value	Cluster size
		*X*	*Y*	*Z*		
*Age^2^*						
lFCD	Frontal_Sup_R	21	12	57	5.91	190
	Hippocampus_L	−36	−9	−24	4.92	367
	Precentral_L	−30	−3	51	4.6	118
	Precuneus_L	−9	−54	51	4.31	71
	Temporal_Inf_R	45	−6	−39	4.03	58
	Caudate_R	12	18	12	4	46
	Postcentral_R	39	−21	48	−3.77	41
FOCA	Temporal_Inf_R	48	−45	−6	5.96	78
	Caudate_R	15	21	15	5.42	197
	Caudate_L	−18	9	24	4.18	152
	Temporal_Inf_L	−57	−27	−21	4.12	510
	Frontal_Inf_Orb_R	42	36	−12	3.97	67
	Cerebelum_6_L	−24	−81	−18	−4.57	908
	Calcarine_R	6	−81	3	−4.11	
	Precentral_L	−42	−12	57	−4.65	77
	Temporal_Sup_L	−57	0	0	−4.03	113
	Postcentral_L	−54	−15	42	−4.03	66
	Heschl_R	54	−12	9	−3.98	59
	Temporal_Sup_R	60	0	0	−3.78	

### 3.2 Relationship between behavior and local FCs

**FIGURE 2:**
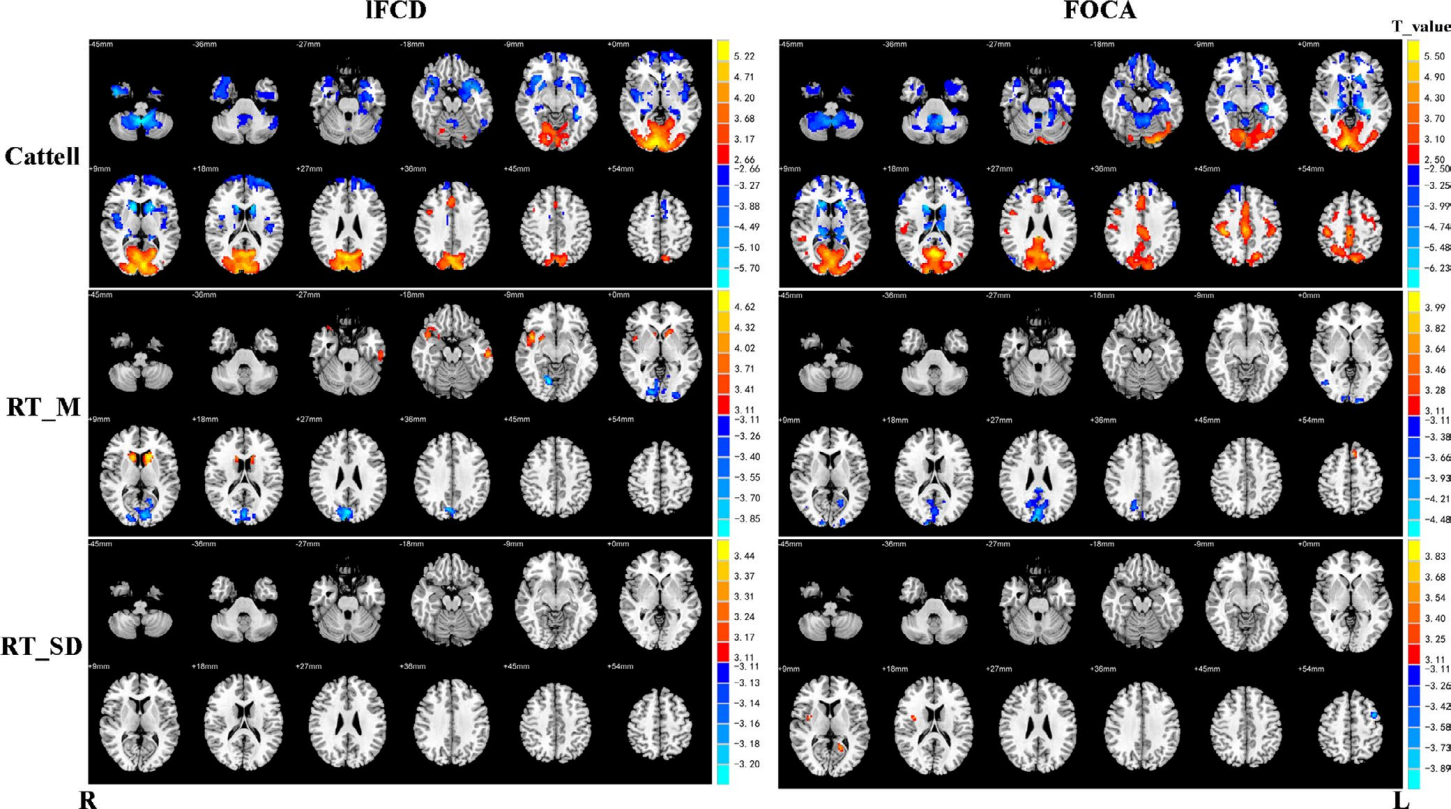
Relationships between local functional connectivity (FOCA and lFCD) and behavior scores (cluster size > 30 voxels; Cattell‐fluid intelligence, *p* < .05, FDR correction; RT_M, RT_SD‐average response time in response time tasks, intraindividual response time variability in response time tasks, *p* < .001, uncorrected). Red areas indicate that local FC decreased significantly as behavior scores decreased, while blue areas indicate the opposite. R stands for the right hemisphere of the brain, and L stands for the left hemisphere of the brain

In order to illustrate the relationship between behavior and local FCs, we calculated the correlation between them. The fluid intelligence (Cattell) was negatively correlated with age in the lifespan while positively related to local FC within DMN, VN and partial SMN. However, there were few significant relationships between local FCs and RT_M/RT_SD (Figure 2). The detailed information could be seen in Table S1.

## 4 DISCUSSION

**FIGURE 3:**
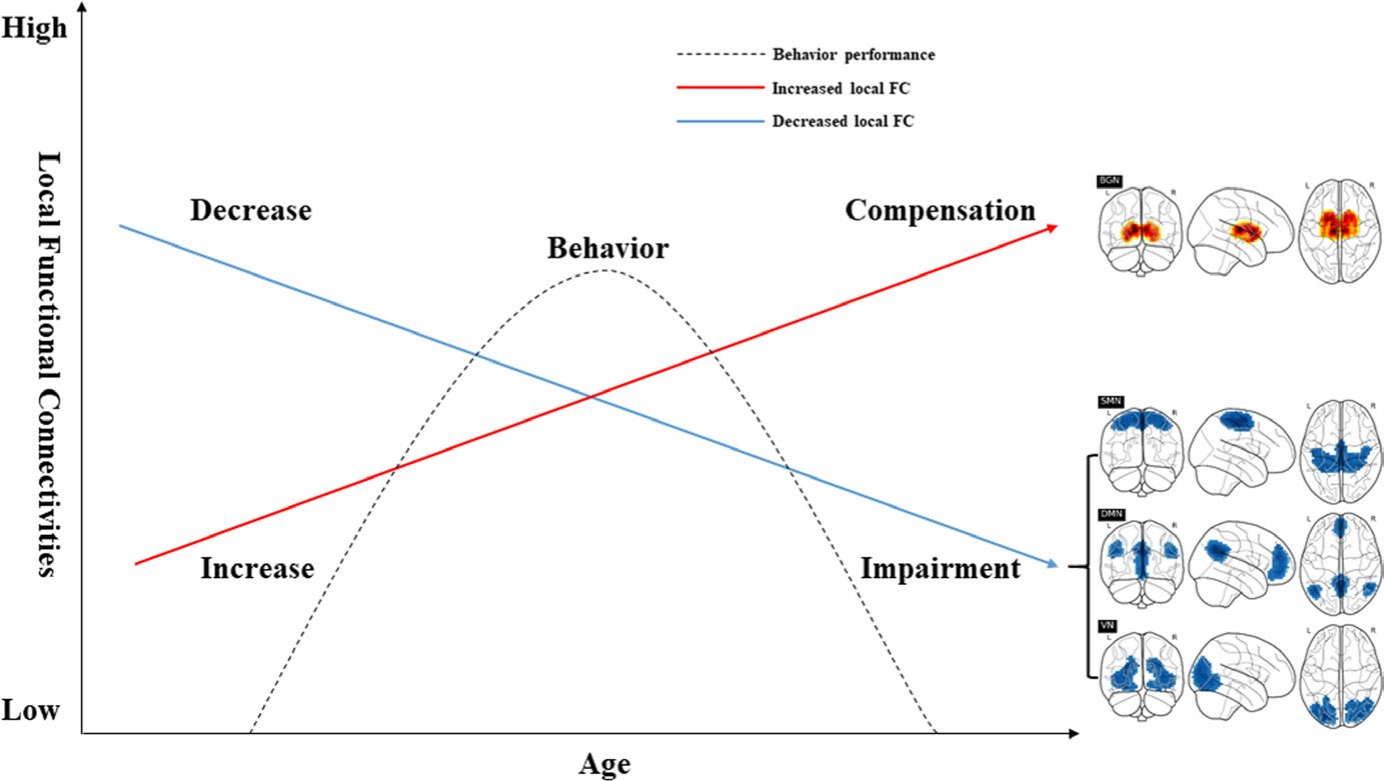
Illustration of the underlying mechanisms of four networks over the lifespan. As age increases, the local FCs within the VN, SMN, and DMN decreased (impairment), while those within the BGN increased (compensation). The behavior scores exhibited an inverted U‐shaped curve across the lifespan

This study explored the alterations in functional networks across the lifespan and the underlying mechanism via local functional connectivity. In brief, as shown in Figure 3, it was found that local functional connectivity (lFCD and FOCA) in the VN, SMN, and DMN decreased across the lifespan while that in the BGN increased across the lifespan. Such results might be explained by the decline in the VN, SMN, and DMN, reflecting the impairment of corresponding functions, while the increase in the BGN indicates compensation for functional networks.

### 4.1 Changes of lFCD and FOCA across the lifespan

The changes of functional connectivity in lifespan have always been a research hot spot, and there have been a few studies on the mechanism behind the changes of functional connectivity. Previous studies have shown that functional connectivity in lifespan decreased with age, mainly within DMN, FPN, CON, SMN, and VN, while some studies suggested that FCs within the partial motor and subcortical networks increased with age (Damoiseaux, 2017; Ferreira & Busatto, 2013). In general, the decline of situational memory, self‐reference processing, and thinking wandering in the elderly leads to the decline of functional connectivity in DMN (Buckner, Andrews‐Hanna, & Schacter, 2008). Moreover, the functional connectivity between DMN and FPN was positively correlated with age, which may be due to the weakening of the separation of the two functional networks with aging (Grady et al., 2016; Spreng et al., 2016). On the other hand, Coste and his colleagues found that the decline in functional connectivity within CON may be due to decreased alertness among older people (Coste & Kleinschmidt, 2016) and Yan, Zhuo, Wang, and Wang (2011) suggested that the decline of VN internal functional connectivity with age may be due to the decline of visual function in the elderly. In addition, the elderly performed an impaired motor performance, yet the consistent changes of FC were not observed within SMN. As the age grows, FC increased within some portions of SMN, while some portions of SMN exhibited the contrary phenomena. The lifespan and aging studies implied that the declines within SMN were associated with decreases in sensory processing and diminished communications within a “sensorimotor feedback” system (Roski, 2013; Seidler et al., 2015). Moreover, fluid intelligence is associated with executive control and intrinsic cognitive ability, which decreases with age growing indicating the elderly might impair with the above abilities (Finn et al., 2015; Gray, Chabris, & Braver, 2003). Our study found that local functional connectivity (lFCD and FOCA) within DMN, VN, and SMN decreased in lifespan. We also found the fluid intelligence was negatively correlated with age in the lifespan while positively related to local FC within DMN, VN, and partial SMN. Together, our results indicate that the decline of fluid intelligence might be linked with decreased local functional connectivity within VN, SMN, and DMN.

### 4.2 The possible compensatory role of the BGN

It was widely believed that the increased FC changes in lifespan could reflect a compensatory mechanism, and most of these changes were intranetwork FC. For example, the FCs between DMN and FPN/attentional networks were increased in lifespan due to the dedifferentiation process of aging brain, occurrence of aging for late neurodevelopmental stages, and a compensatory for cognitive decline (Grady et al., 2016; Zhai & Li, 2019; Zonneveld et al., 2019). Meanwhile, the BGN was thought to be related to motor learning functions and the increased FC within BGN might suggest as a compensatory role of dysfunctions of motor learning, especially in Parkinson's disease‐related research (Lyman, Anguera, & Terman, 2011; Saling & Phillips, 2008). Furthermore, it was also found that the homologous basal ganglia circuitry was related to cognition and emotion (Saling & Phillips, 2008) and an increased FC within it might compensate high‐order cognition (Siman‐Tov, 2016). In lifespan, the BGN might play a compensatory role to balance the dysfunction of other functional networks. In our study, the local FC (lFCD and FOCA) with BGN increased in lifespan reflects that the increased FCs in BGN might provide the compensatory for the decline function networks of primary and high‐order cognition. In summary, the BGN might regulate other functional networks (VN, SMN, and DMN) and compensate the corresponding cognitive decline in lifespan.

### 4.3 Limitations

Although our findings could give insight into the compensatory role of the BGN across the lifespan to some degree, several limitations should be addressed. First, behavioral data were relatively insufficient. Further research should involve more behavioral data to reveal more detailed cognitive relationships between local FCs, such as working memory, motor ability, and emotion recognition. Second, fMRI has low temporal resolution compared to electroencephalogram (EEG). In the future, EEG data should be involved to study real‐time effective behavior results. Finally, because of the accurate compensatory effects are not tested in this work, the accurate mechanism of the BGN remains unclear. Our study perhaps offers one possible explanation of the compensatory mechanism of the BGN across the lifespan through local FCs, and more research efforts are needed in this regard. Additionally, since the distribution of functional hubs in the human brain is a critical topic, it would be helpful to expand our research with complex network analysis in future work.

## 5 CONCLUSION

In this study, we used resting‐state lFCD and FOCA measures to investigate the changes of local functional connectivity in lifespan and tried to give the underlying mechanism: The decline of cognitive function in the aging population led to the decline of FC in VN, SMN, and DMN. Meanwhile, BGN may play a regulatory role in the aging process to compensate for the dysfunction of other functional systems.

## CONFLICT OF INTEREST

All authors have no conflicts of interest to disclose.

## AUTHOR CONTRIBUTIONS

LD and JC conceived the project. XW, HH, and LD implemented the statistical analysis. JY preprocessed the data. XW, HH, and LD wrote and revised the paper. HH, DY, HG, and CL provided critical suggestions for the manuscript.

## Supporting information

Supplementary Material

## Data Availability

Publicly available datasets were analyzed in this study. This data can be found here: https://camcanarchive.mrc‐cbu.cam.ac.uk/dataaccess/.
