# Supplementary material for: Alterations of local functional connectivity in lifespan: A resting‐state fMRI study
Source: Brain Behav. 2020 May 27;10(7):e01652. doi: 10.1002/brb3.1652 (PMC7375100; doi:10.1002/brb3.1652)
Supplement: Supplementary file 1 — Supplementary Material [file BRB3-10-e01652-s001.docx]

Table S1 Detailed information on each brain region in the relationship between local FC and behavior score

|  | index | region | MNI coordinates | | | peak t_value | cluster size |
| --- | --- | --- | --- | --- | --- | --- | --- |
|  |  |  | X | Y | Z |  |  |
| FCD | Cattell | Temporal_Inf_L | -57 | -54 | -21 | -3.7529 | 239 |
|  |  | Cerebelum_4_5_L | -6 | -57 | -18 | -3.5515 | 71 |
|  |  | Rectus_L | 0 | 24 | -24 | -3.0274 | 39 |
|  |  | Calcarine_R | 9 | -90 | 0 | 5.7271 | 4367 |
|  |  | Cingulum_Post_R | 3 | -36 | 27 | 3.9863 | 32 |
|  |  | Frontal_Mid_R | 42 | 6 | 36 | 3.5461 | 49 |
|  |  | Cingulum_Mid_R | 3 | 21 | 39 | 4.1389 | 120 |
|  |  | Supp_Motor_Area_L | -6 | 18 | 54 | -3.9679 | 104 |
|  | RT_M | Cerebelum_8_R | 36 | -45 | -54 | 4.9278 | 88 |
|  |  | Temporal_Mid_L | -63 | -21 | -18 | 4.4993 | 59 |
|  |  | Insula_R | 42 | 6 | -12 | 4.5053 | 129 |
|  |  | Putamen_R | 30 | -3 | -6 | 4.4039 | 49 |
|  |  | Cuneus_R | 6 | -90 | 27 | -3.9974 | 512 |
|  |  | Caudate_L | -15 | 18 | 9 | 4.8572 | 134 |
|  |  | Caudate_R | 15 | 15 | 9 | 4.6132 | 73 |
|  |  | Parietal_Sup_L | -21 | -42 | 72 | 3.7716 | 49 |
| FOCA | Cattell | Hippocampus_L | -36 | -30 | -6 | -6.9713 | 6737 |
|  |  | Calcarine_L | 0 | -66 | 18 | 6.1016 | 6024 |
|  |  | Temporal_Mid_R | 51 | -45 | -6 | -3.3293 | 30 |
|  |  | Temporal_Sup_R | 48 | -39 | 9 | 3.3965 | 37 |
|  |  | SupraMarginal_R | 57 | -27 | 21 | 3.3353 | 67 |
|  |  | Precentral_R | 45 | 6 | 33 | 4.1401 | 72 |
|  |  | Postcentral_R | 39 | -30 | 48 | 5.4306 | 362 |
|  |  | Parietal_Inf_L | -45 | -27 | 45 | 3.8851 | 207 |
|  | RT_M | Cerebelum_7b_R | 45 | -57 | -57 | 4.1703 | 65 |
|  |  | Cuneus_R | 6 | -90 | 27 | -4.7602 | 293 |
|  |  | Calcarine_L | -18 | -60 | 12 | -3.9196 | 30 |
|  | RT_SD | Precentral_L | -39 | 0 | 57 | -4.0425 |  |

Table S1 illustrated the detailed relationship between local FCs and behavior scores (Cattell, RT_M, and RT_SD). Noteworthy, there was none significant relationship between FCD and RT_SD.

Table S2 Information of participants

|  |  | number | Male/Female | Range (years/values) | Mean | SD |
| --- | --- | --- | --- | --- | --- | --- |
| Age | ALL | 523 | 258/265 | 18-88 | 51.28107 | 17.6018 |
|  | Younger | 215 | 103/112 | 18-45 | 33.84651 | 6.9188 |
|  | Middle | 172 | 81/91 | 46-65 | 54.6163 | 5.9125 |
|  | Old | 136 | 74/62 | 66-88 | 74.625 | 5.7342 |
| Cattell | ALL | 509 | 254/255 | 12-44 | 32.8271 | 6.3794 |
|  | Younger | 207 | 101/106 | 22-22 | 36.6087 | 4.3502 |
|  | Middle | 170 | 81/89 | 18-42 | 33.1588 | 4.7543 |
|  | Old | 132 | 72/60 | 12-39 | 26.4697 | 5.9294 |
| RT_M | ALL | 476 | 236/240 | 0.3582-1.1197 | 0.5751 | 0.1314 |
|  | Younger | 197 | 95/102 | 0.3582-0.9113 | 0.3686 | 0.0763 |
|  | Middle | 157 | 75/82 | 0.4223-0.9361 | 0.5835 | 0.1003 |
|  | Old | 122 | 66/56 | 0.4325-1.1197 | 0.6979 | 0.1371 |
| RT_SD | ALL | 476 | 236/240 | 0.0435-0.3980 | 0.1292 | 0.0646 |
|  | Younger | 197 | 95/102 | 0.0435-0.2704 | 0.9545 | 0.0379 |
|  | Middle | 157 | 75/82 | 0.0516-0.3018 | 0.1282 | 0.0519 |
|  | Old | 122 | 66/56 | 0.0731-0.3981 | 0.1849 | 0.0744 |

To illustrate the relationship between age and Cattell score, a scatter plot with a linear fitting line (the corresponding R^2^ = 0.43, p_value=3.6e^-63^) was shown in Figure S1 indicating that age and Cattel score is negatively correlated. Because the changes of mean FCs across lifespan was mainly linear and quadratic (the Inverted U shape) (Wei et al., 2018) and FC changes within some functional networks (VN, CEN) have linear decreases across lifespan while those within DMN have quadratic (the inverted U shape) decreases, and SMN, SAN shown both linear and quadratic decreases of FCs (Vij et al., 2018; Wang et al., 2012). It is reasonable to apply a GLM to reveal the age effect on local FCs. Furthermore, the age and behavioral score were strongly correlated (r^2^ = 0.43, p = 3.6e^-63^). If the behavioral score was added in the GLM in Eqn. (1), it would lead to a collinearity problem. And, according to the previous studies (Kievit et al., 2014; Onoda et al., 2012), we used two GLM to consider these two measures respectively.


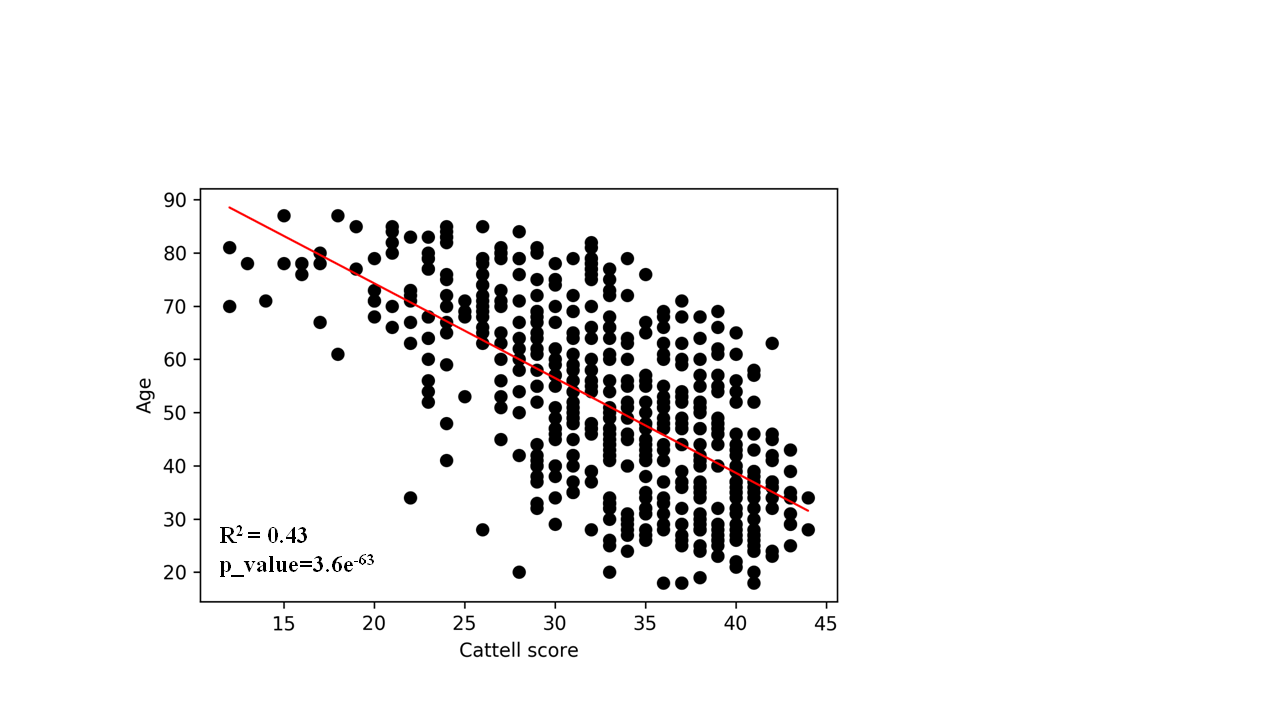


Figure S1 Relationship between Cattell score and age. Redline indicated the linear regression fitting for Cattell score and age, the corresponding R^2^ = 0.43 and p_value=3.6e^-63^.

The lFCD with time course (TC) threshold from 0.5 to 0.7, step size = 0.1 were also calculated to explore whether the different thresholds of TC have an impact on the results. There are small differences among different TC threshold (0.5, 0.6, 0.7), as expected, TC = 0.5 leads to increased false-positive rate and TC =0.7 lead to decreased false positive rate compared to TC = 0.6. (Figure S2 and Figure S3) However, the main results remained the same with ones in the paper: the lFCD values in the olfactory cortex, the superior temporal gyrus, right insula, hippocampus, right amygdala, cerebellum inferior and right caudate nucleus was positively correlated with age. While areas that negatively correlated with IFCD involved occipital gyrus, calcarine, left cuneus, left lingual gyrus, inferior frontal gyrus, left precentral gyrus and left medial superior frontal gyrus. At the same time, the FOCA values of thalamus, caudate nucleus, hippocampus, superior temporal gyrus, left middle frontal gyrus and left medial superior frontal gyrus were positively correlated with age; the areas which negatively correlated with FOCA involved right calcarine, right paracentral lobule, right lingual gyrus, cerebellum superior, cerebellum superior, right medial and lateral cingulate gyrus, postcentral gyrus and left superior orbital frontal gyrus. The results of age^2^ are similar to those in the paper when applying the TC threshold to 0.5. None significant result is found when applying the TC threshold to 0.7.


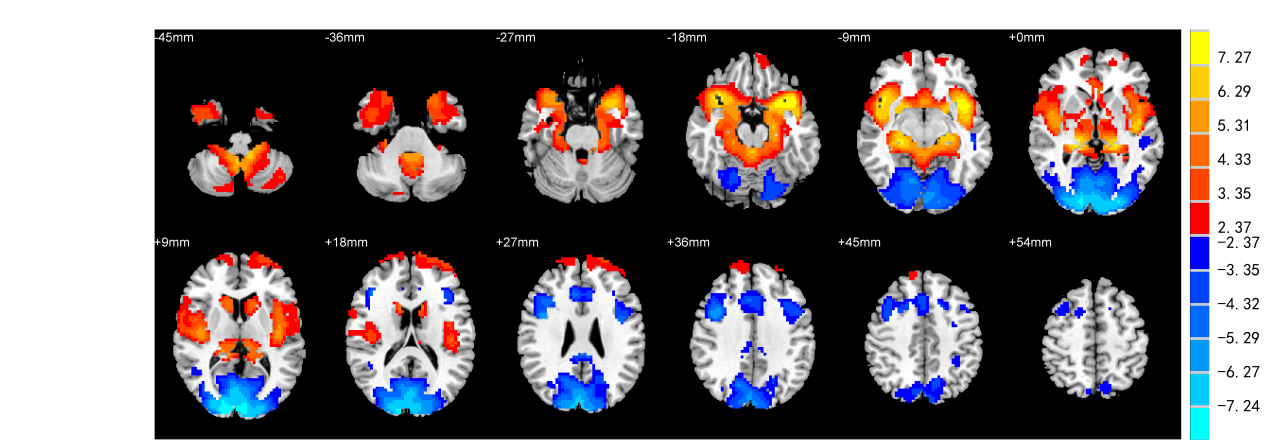


Figure S2. Relationships between local functional connectivities (lFCD, threshold = 0.5) and age. (cluster size>30，p<0.05，false discovery rate (FDR) correction). Red areas indicated that local FC increased significantly as age growing while blue areas indicated the contrary.


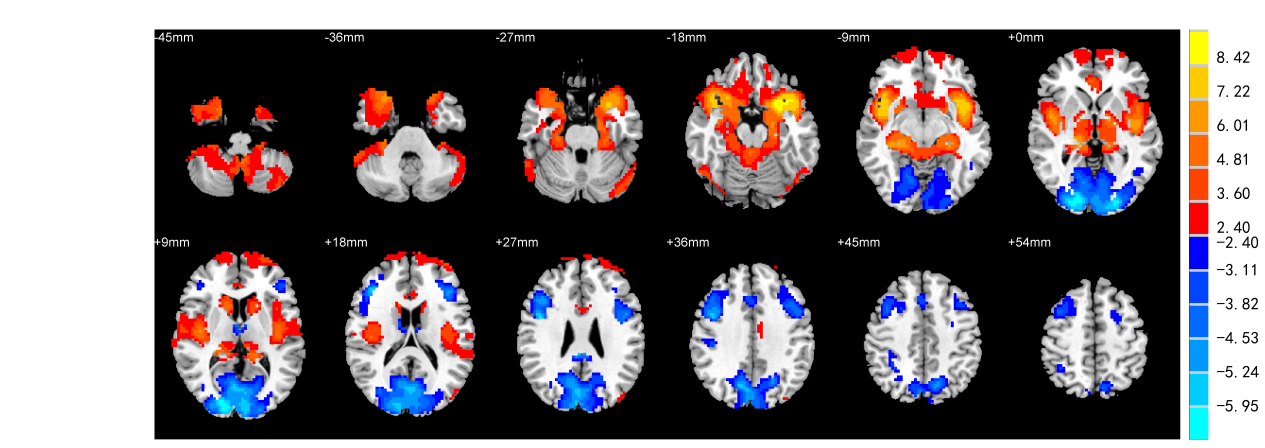


Figure S3. Relationships between local functional connectivities (lFCD, threshold = 0.7) and age. (cluster size>30，p<0.05，false discovery rate (FDR) correction). Red areas indicated that local FC increased significantly as age growing while blue areas indicated the contrary.

This work focus on the changes of local FC across the lifespan, while not the topology properties computed by complex network analysis. Considering the distribution of functional hubs in the human brain is a critical topic, here, we calculated the degrees of global functional connectivity networks at voxel level (with correlation threshold of 0.6) to preliminarily show the changes of functional hubs across lifespan in the brain. The results showed that the degree in the Temporal_Pole_Sup_L\R, ParaHippocampal_L\R, Amygdala_L\R, Lingual_L\R, Hippocampus_L\R, Insula_L\R, Caudate_L\R, Putamen_L\R, Pallidum_L\R, Temporal_Sup_L\R and Thalamus_L\R were positively correlated with age. While areas that negatively correlated with age involved Occipital_Sup_L\R, Cingulum_Post_L\R, Parietal_Inf_L\R, Frontal_Sup_L\R, Angular_L\R, Parietal_Sup_L\R, Frontal_Sup_Medial_L\R, SupraMarginal_L\R, Precuneus_L\R, Cingulum_Mid_L\R, Frontal_Inf_Oper_L\R, Frontal_Mid_L\R, Cuneus_L\R, Rolandic_Oper_L\R, Occipital_Mid_L\R, and Calcarine_L\R. Additionally, the degree of all voxels in the brain in the Fusiform_L, Hippocampus_L, Putamen_L\R, Caudate_L\R, Frontal_Inf_Oper_R, Cingulum_Post_L\R, and Frontal_Sup_R were quadratically increased across the lifespan while those in the Postcentral_R shown the quadratically decreases across the lifespan (Figure S4).


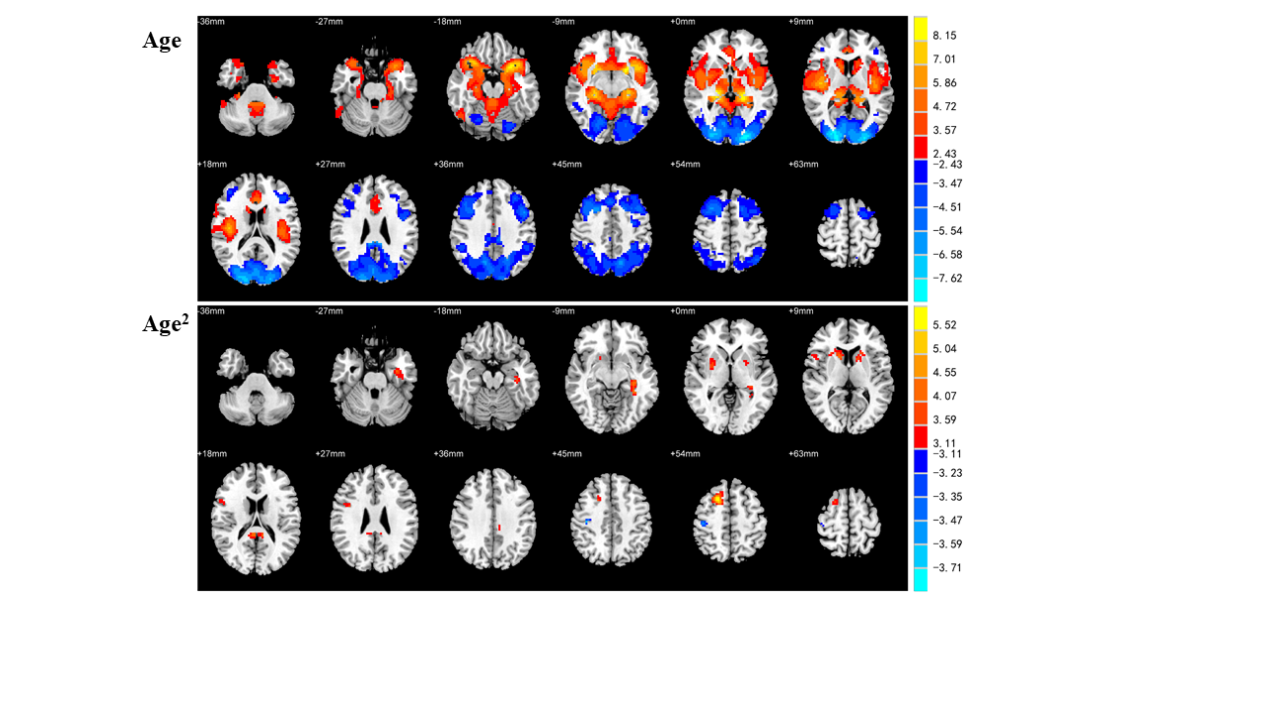


Figure S4. Relationships between the degree of all voxels in the brain and age/age^2^. a) impact of age on the degree of all voxels in the brain (cluster size>30，p<0.05，false discovery rate (FDR) correction), b) impact of age^2^ on the degree of all voxels in the brain (cluster size>30，p<0.01，uncorrected). Red areas indicated that the degree of all voxels in the brain increased significantly as age growing while blue areas indicated the contrary.

Scheinost et al., have done a work of sex differences in normal age trajectories of functional brain networks (Scheinost et al., 2015). In their paper, a variate of age × gender was added to the GLM to measure the impacts of age and gender together. Therefore, here, we analyzed gender and age influences to local FC inspired by Scheinost’s research (equation R1). As Figure S5 illustrates, gender and age differences have few impacts on local FC in our study. The lFCD values in the Temporal_Inf_R, Temporal_Pole_Sup_R and Hippocampus_R have a greater slope associated with age for females compared with males. While those in the Supp_Motor_Area_L have greater slope associated with age for males compared with females. Meanwhile, the FOCA values in the Cerebelum_Crus2_L\R, Cerebelum_4_5_R, Vermis_6, and Temporal_Inf_L\R have greater slope associated with age for female compared with male. While those in the Frontal_Inf_Tri_L\R, Temporal_Mid_L\R, Rolandic_Oper_L\R, Postcentral_R, Precentral_L\R, Precuneus_R, Supp_Motor_Area_L and Fusiform_R have greater slope associated with age for male compared with female. And the corresponding results are added to the supplementary material.

Equation S1:

$localFC=\beta_{0}+\beta_{1}\times age+\beta_{2}\times age^{2}+\beta_{3}\times X_{sex}+\beta_{4}\times X_{sex \times age}+\beta_{5}\times X_{mFD}+\beta_{6}\times X_{volume}$


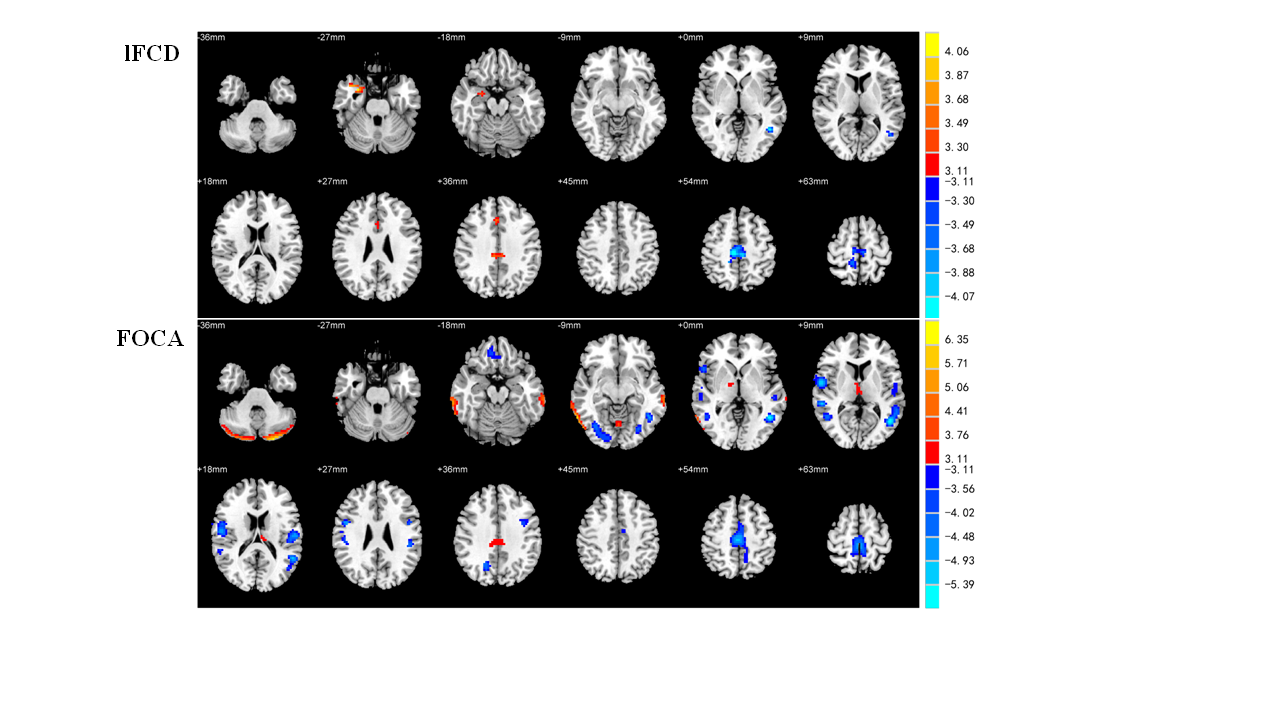


Figure S5. Relationships between local functional connectivity (lFCD, threshold = 0.6 and FOCA) and gender. (cluster size>30，p<0.001，uncorrected). Red areas indicated that local FC was greater slope associated with age for female compared with the male while blue areas indicated that local FC was greater slope associated with age for female compared with male.

References:

Kievit, R.A., et al., 2014. Distinct aspects of frontal lobe structure mediate age-related differences in fluid intelligence and multitasking. Nat Commun. 5**,** 5658.

Onoda, K., Ishihara, M., Yamaguchi, S., 2012. Decreased functional connectivity by aging is associated with cognitive decline. J Cogn Neurosci. 24**,** 2186-98.

Scheinost, D., et al., 2015. Sex differences in normal age trajectories of functional brain networks. Hum Brain Mapp. 36**,** 1524-35.

Vij, S.G., et al., 2018. Evolution of spatial and temporal features of functional brain networks across the lifespan. Neuroimage. 173**,** 498-508.

Wang, L., et al., 2012. Decoding lifespan changes of the human brain using resting-state functional connectivity MRI. PLoS One. 7**,** e44530.

Wei, D., et al., 2018. Structural and functional brain scans from the cross-sectional Southwest University adult lifespan dataset. Sci Data. 5**,** 180134.
